# The role of teleradiology during COVID-19 outbreak: Saudi radiologists’ perspectives

**DOI:** 10.15537/smj.2023.44.2.20220793

**Published:** 2023-02

**Authors:** Shrooq T. Al-Dahery, Walaa M. Alsharif, Fatima H. Alamri, Shahad A. Nawawi, Wed K. Mofti, Fahad H. Alhazmi, Khalid M. Alshamrani, Awadia G. Suliman, Abdulaziz A. Qurashi

**Affiliations:** *From the Department of Applied Radiologic Technology (Al-Dahery, Alamri, Nawawi), College of Applied Medical Sciences, and from the Faculty of Medicine (Mofti), University of Jeddah; from the College of Applied Medical Sciences (Alshamrani), King Saud bin Abdulaziz, University for Health Sciences; from the Department of Radiological Sciences (Alshamrani). King Abdullah International Medical Research Center; from the Ministry of the National Guard - Health Affairs (Alshamrani), Jeddah; and from the Diagnostic Radiology Technology Department (Alsharif, Alhazmi, Suliman, Qurashi), College of Applied Medical Sciences, Taibah University, Al Madinah Al Munawwarah, Kingdom of Saudi Arabia.*

**Keywords:** teleradiology, radiology, COVID-19, communication, patient’s confidentiality, image interpretation

## Abstract

**Objectives:**

To evaluate the role of teleradiology during the COVID-19 pandemic from Saudi radiologists’ perspectives to improve the radiology quality service.

**Methods:**

A cross-sectional study was carried out in Saudi Arabia among radiologists working at local hospitals from October to November 2021. It contains 21 questions involved demographic information; general information on teleradiology services; and the impact of teleradiology during COVID-19. One-way ANOVA was used to compare demographic groups. Chi-square test was used to compare demographic groups regarding their distribution of responses. All tests were carried out <0.05 level of significance.

**Results:**

A total of 102 radiologists participated in this study (56% males, 44% females), 58.8% of them were sub-specialized in chest radiology. Regarding the general status of teleradiology, 69.6% of participants believed that teleradiology is a helpful tool for imaging interpretation. However, 44% of them were uncertain on the impact of teleradiology on patients’ confidentiality. Approximately 87% of participants agreed that there is a positive contribution of teleradiology during COVID-19, which enables decreasing risk of infection and workload. There was a significant difference between professional degrees and overall participant responses (*p*<0.05). Academicians agreed that it enhances radiology departments’ work (mean=17.78, SD=1.86).

**Conclusion:**

Concerns raised on complicated cases that require physical presence of patients, cannot be performed by teleradiology. Additionally, it might provide insufficient communication with other professionals to discuss images.

**C**oronavirus disease (COVID-19) was declared a global pandemic in December 2019, and maintaining the healthcare sector has been one of the most important priorities in protecting healthcare professionals and patients.^1^ The COVID-19 pandemic has posed substantial pressure on healthcare systems around the world, including those in Saudi Arabia. All countries have experienced a flow of critically infected patients with COVID-19, resulting in the spread of infection among healthcare workers, as well as a lack of resources (such as personal protective equipment) to cope with the disease.^2^ Saudi Arabia is similar to other countries that has faced challenges in delivering healthcare services. This pandemic affected a large population worldwide. Lifestyle changes lead to high incidences of chronic disorders such as type II diabetes mellitus and heart disease, which is compounded by increasing expenditures, patient expectations, and workloads of healthcare staff and facilities.^3^ According to the National Transformation Program (2030 Vision), the Ministry of Health (MOH) in Saudi Arabia aims to improve the standards, equitability, availability, and quality of healthcare across the country by activating the use of electronic communication and information technology (IT) in the healthcare sector.^4^ A considerable amount of the Saudi government’s budget has been allocated to the healthcare sector, making activating digital health solutions a high priority, particularly during the COVID-19 pandemic.^5-8^ Proactive measures have been taken to meet the needs of the community by using advanced methods, such as the use of teleradiology in radiology departments.^9^

Providing healthcare services remotely through teleradiology has been an important aspect of telemedicine, as it can enhance service efficiency.^10^ A previous study was carried out by Gupta et al,^8^ 2020, found that teleradiology is a useful tool since it fosters better peer collaboration. In addition, Alshamrani and Alkenawi,^6^ 2021 have reinforced the necessity of IT integration services for improving radiology practices.Teleradiology is a medical information system that requires the use of telecommunication systems (such as internet, mobile phones, and computers) in order to exchange patient data, images, and other radiological information to ensure secure radiology services from site to site and remotely at any time.^11^ It tends to be used to resolve the overnight workloads of radiology services, deal with urgent cases, solve radiologist shortage issues, and provide remote consultation via an off-site or sub-specialized radiologist. In addition, teleradiology can provide some administrative aspects such as technical support and quality control.^9^

Due to the COVID-19 pandemic, healthcare resources worldwide, especially radiology departments, have been directed toward the care of COVID-19 patients. Many healthcare personnel, including radiologists, have been infected, and this added pressure on healthcare systems.^12^ Also, this may disturb the daily working and personal life of radiology personnel. Several studies emphasized the influence of the COVID-19 crisis on radiologists’ life (such as profession, physiological wellbeing).^13,14^ Physiological issue was predominant among frontline healthcare workers including radiologists due to their direct engagement with COVID-19 diagnosis and treatment.^13^ The prevalence of depression, insomnia, and anxiety among radiologists was also highlighted in the literature. This was reinforced later by Coppola et al,^14^ who claimed that radiologists were anxious of getting infected and spreading the infection among their families and colleagues, which may negatively impact their professional and social relationships. Therefore, in many radiology departments, the enhancement of teleworking solutions has become more prevalent to cope with the COVID-19 pandemic.^15^ However, the shift to radiologists working remotely is complex due to the need for sophisticated technology, such as a radiology information system (RIS) and picture archive and communication system (PACS), to successfully operate the teleradiology system.^15,16^

Although other countries have shown that teleradiology improves peer-to-peer collaboration, organizing radiologists’ workloads, and thus enhancing the quality and efficiency of radiology services.^11^ To the best of the investigators’ knowledge, this is the first study to assess the role of teleradiology in radiology departments during COVID-19 from the perspective of Saudi radiologists.

## Methods

The University of Jeddah’s Bioethics Committee of Scientific and Medical Research granted ethical approval for this study (Reference Number: HAP-02-J-094 – Application number: UJ-REC-028). All participants were informed of the study’s nature, their participation in the study was voluntary, and confidentiality was maintained. Written consent was obtained from the participants who agreed to participate in the study.

A cross-sectional study design was applied in this study. An online questionnaire was distributed among radiologists working in local hospitals in Saudi Arabia between October and November 2021 by the study researchers. The questionnaire was adapted from the following previously published articles with minor modifications and contained 21 closed-ended questions covering the following 3 main sections: i) demographic background information; ii) general information on teleradiology services; and iii) the impact of COVID-19 on the usage of teleradiology services.^18-20^ The subjective nature of the questions was a key factor in designing the questionnaire, providing an understanding of the impact of teleradiology during the pandemic from a radiologist’s point of view. Using the MeSH Advanced Search Builder of Pubmed, we searched for resources that met the inclusion criteria by combining the following keywords: teleradiology, radiology, COVID-19, communication, patient’s confidentiality, and image interpretation. Studies published before 2016 were excluded.

A web-based questionnaire created through Google Forms was utilized for this study and sent to radiologists working in Saudi Arabia using non-probability convenient sampling technique.^21^ A pilot study was carried out with 3 academic lecturers and radiologists with experience varying from 5 to 10 years. Minor corrections were made to the questionnaire based on the pilot study feedback received. The recruitment process included an invitation to participate in this study, which was sent out via a variety of online portals including social media applications such as WhatsApp and Twitter. This invitation indicated the goals of the research in brief and provided information on the research team; permission was also facilitated for each participant. The inclusion criteria involved radiologists working at local hospitals in Saudi Arabia.

### Statistical analysis

Data was analyzed using the SPSS statistical package, version 26 (IBM SPSS, Chicago, IL, USA), and a *p*-value of <0.05 was considered significant. An overall evaluation of the teleradiology status was measured through a score that was calculated per participant. These scores were separately calculated for the general teleradiology questions and the questions regarding teleradiology in the COVID-19 context. To compare demographic groups in terms of the scores, one-way ANOVA was used. A Chi-square test was also used to compare demographic groups in terms of the distribution of responses to each individual question.

## Results

A total of 102 radiologists agreed to participate (56% males and 44% females). Most of the participants (85%) were either radiology residents or consultant radiologists (44% and 41%, respectively). The demographic characteristics of the years of experience, subspecialty, professional degree, sector, and utilization of teleradiology are shown in Table 1.

**Table 1:** - Demographic characteristics of Saudi radiologists (N=102).

Variable	n	%
***Gender***		
Male	57	55.9
Female	45	44.1
***Experience (years)***		
1-5	44	43.1
6-10	26	25.5
>10	32	31.4
***Subspecialty***		
Chest radiology	60	58.8
Women imaging	13	12.7
Cardiovascular imaging	8	7.8
Other*	21	20.6
***Professional degree***		
Radiology resident	45	44.1
Consultant radiologist	42	41.2
Associate professor	9	8.8
Professor	6	5.9
***Sector***		
Private hospitals	50	49.0
Semi-public/military/national guard hospitals	16	15.7
Public hospitals	32	31.4
Academic hospitals	4	3.9
***Utilization of teleradiology in the participant’s workplace***		
Utilized	31	30.4
Unutilized	71	69.6

Values are presented as number and percentage (%).

^*^ Including: neuroradiology, musculoskeletal, abdomen, vascular and interventional radiology, pediatric radiology, nuclear radiology and general radiology.

**Figure 1:**
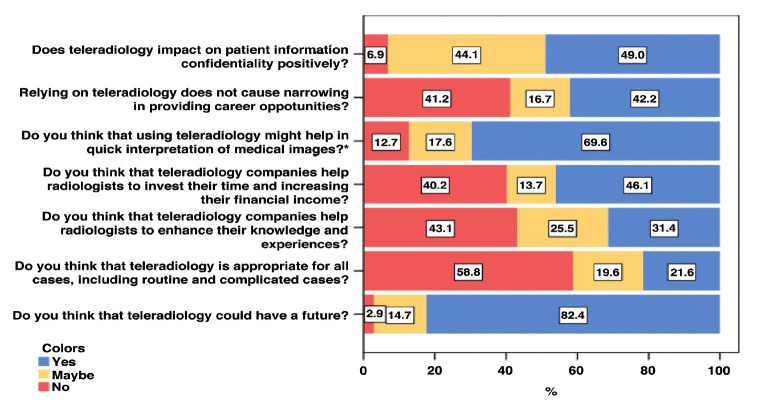
- The general status of teleradiology. *Of those who said “yes” (n=71), 4.2% reported that the expected speed ratio for the reporting period compared to the traditional method is “5%,” 19.7% reported that the speed is “10%,” 33.8% reported that the speed is “20%,” 19.7% reported “40%”, and 22.5% reported other percentages.

Regarding the general status of teleradiology, the results revealed that most of the participants (69.6%) agreed that teleradiology is a helpful tool in terms of imaging interpretation. In addition, 49% of participants agreed that the use of teleradiology would increase without a negative impact on patient confidentiality. However, 44% of the participants were uncertain on the impact of teleradiology on patient privacy and confidentiality. Additionally, over half of the participants (59%) believed that teleradiology is inappropriate for all examination types, such as complicated cases (Figure 1).

No association was found between hospital types, years of experience, and the overall participants’ responses during the COVID-19 pandemic (*p*>0.05). However, a significant difference was found between the participants’ professional degrees and the overall participants’ responses (*p*<0.05) (Table 2). Academic participants showed greater agreement (mean=17.78, SD=1.86) on the role of teleradiology systems in enhancing the work of radiology departments (Table 2).

**Table 2:** - The association of demographic variables and the evaluation of teleradiology in general status (overall score).

Demographic variable		Using the raw scores		*P*-value
	n	Mean	SD	
***Work experience***				
1-5	44	14.86	3.43	0.336
6-10	26	15.38	3.24	
> 10	32	16.03	3.44	
***Professional degree***				
Radiology resident	45	15.78	3.35	0.032
Consultant radiologist	42	14.64	3.07	
Associate professor	9	17.78	1.86	
Professor	6	13.67	5.54	
***Sector***				
Private sector	50	14.80	3.31	0.072
Semi-public/military/national guard sector	16	15.44	3.08	
Public sector	32	16.50	3.40	
Academic sector	4	13.00	3.74	

**Figure 2:**
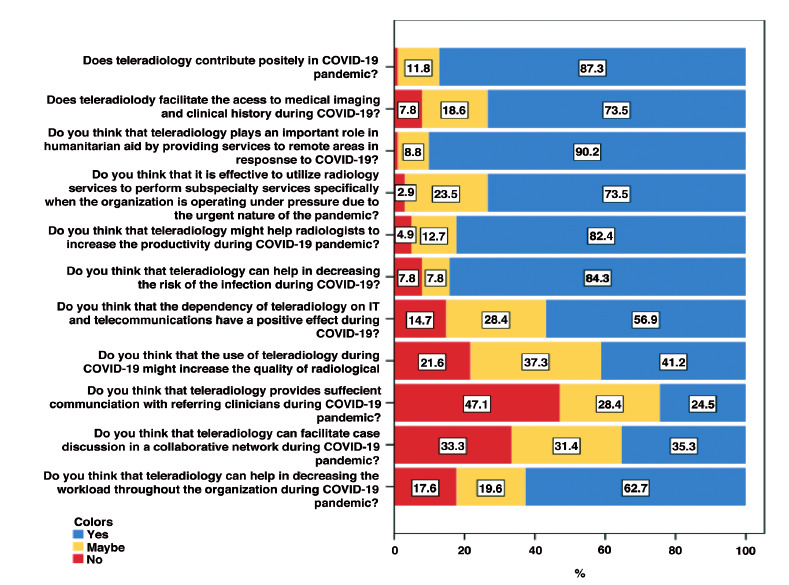
- The Status of teleradiology during COVID-19 pandemic.

Regarding the role of teleradiology throughout the COVID-19 pandemic, the results demonstrated that most participants agreed that teleradiology made a positive contribution in terms of remote access to medical imaging and clinical history. Furthermore, most of the study participants agreed that teleradiology could increase radiologists’ productivity and decrease the risk of infection as well as workload. Half of the participants either did not agree or were uncertain regarding the effect of teleradiology on report quality during the pandemic, while approximately 25% of them thought that teleradiology could provide sufficient communication with other healthcare professionals (Figure 2).

No association was found between professional degree, years of experience, and participants’ overall responses on the role of teleradiology during COVID-19 (*p*>0.05). However, a significant difference was found between hospital types and patients’ overall responses (*p*<0.05) (Table 3). Those working in public hospitals showed higher agreement (mean=28.28, SD=3.07) regarding the role of teleradiology systems during the pandemic (Table 3).

**Table 3:** - The association of demographic variables and the evaluation of teleradiology during the COVID-19 pandemic (overall score).

Demographic variable	Using the raw scores			*P*-value
	n	Mean	SD	
***Work experience***				
1-5	44	27.09	3.20	0.451
6-10	26	27.58	2.12	
>10	32	28.06	4.14	
***Professional degree***				
Radiology resident	45	28.04	3.64	0.354
Consultant radiologist	42	26.93	3.15	
Associate professor	9	28.22	1.64	
Professor	6	26.67	3.33	
***Sector***				
Private hospitals	50	27.64	2.95	0.002
Semi-public/military/national guard hospitals	16	27.06	2.79	
Public hospitals	32	28.28	3.07	
Academic hospitals	4	21.75	6.08	

## Discussion

The aim of the study was to explore the role of teleradiology systems in monitoring workflow during the COVID-19 pandemic in Saudi Arabia. To the best of the authors’ knowledge, this is the first study to explore the perceptions of teleradiology during COVID-19 among Saudi radiologists.

Sixty percent of the study participants sub-specialized in chest radiology, suggesting the importance of teleradiology when dealing with COVID-19 due to its association with thoracic pathologies. In Saudi Arabia, telemedicine, including teleradiology, is becoming a recognized approach to healthcare service delivery. However, the use of teleradiology is still below expectations due to a variety of factors such as regulations, licensing, and financial issues.^9^

Teleradiology involves the electronic transfer of medical imaging from one hospital to another or from one geographical region to another for the purpose of patient diagnosis.^6^ This raises important concerns regarding ethics and clinical practice in terms of the confidentiality of patient information, privacy, and technological reliability.^22^ Although teleradiology is now well established in some countries, and several technical assessments have been applied to this technology, the legal and ethical considerations remain unclear.^23^ Technical support is crucial in order to maintain data security and patients’ privacy by providing proper information protection with the highest level of security to all users.^23,24^ A study by Martin-Noguerol et al^15^ (2020) agreed that clinical data and patients’ images should only be accessible to the reporting radiologists. Appropriate training is required for all teleradiology users to overcome both technical issues and confidentiality considerations.^15^

Similar opinions were expressed by the participants related to the positive effects of teleradiology on career opportunities, time, financial income, knowledge, and experience. In contrast, a small group of the study participants expressed negative opinions. This may lead to questions on whether those radiologists would prefer in-house reporting rather than interpreting images off-site, or whether they may not yet be familiar with teleradiology systems or have used teleradiology in their clinical practice and thus could not express real opinions on their experiences. This result was consistent with the results of the study carried out by Goelz et al^22^ (2021), who found that some radiologists have concerns related to the speed of communication, the equality of reports, residents’ training, the instability of the job market, and radiologists’ incomes.

A similar study by Rosenkrantz et al^18^ (2019) reported that teleradiology can assist clinicians in covering wider geographical locations, as well as facilitate multispecialty practice coverage. However, 59% of the radiologists indicated that teleradiology is not appropriate for all cases, particularly those that are complicated. This may be because although teleradiology relies on static images for diagnosis, and patients are not required to be present physically during the image interpretation, using this technology remains difficult in some cases, such as with complicated ultrasound scans, angiograms, biopsies, pediatric radiology, or interventional radiology.^25^ Additionally, some radiologists may face difficulty in image interpretation via teleradiology due to the insufficient integration of patient history or previous examination, as well as the lack of communication with the referral clinicians.^22^ The results showed that associate professors were more satisfied with the role of teleradiology during the COVID-19 pandemic than others, which may stem from the current practice of those who work in the academic field, as teleradiology systems allow for hybridized academic practice, in which academicians can have a part-time non-teaching clinical role via teleradiology in addition to their role as a teacher.^7^ The potential selection bias (such as imbalance between clinical radiologists and academicians in the survey population) is attributed to the use of a non-probability convenient sampling technique, since the research question addressed by this technique is limited to the study population itself and thus lacks generalizability among clinical radiologists and academicians. Therefore, this result needs to be treated with caution due to the small sample size.

In terms of the role of teleradiology systems during the COVID-19 pandemic, most of the participants in the current study agreed on the positive impact of teleradiology in facilitating access to healthcare services, patients’ clinical information, and providing services to remote areas. This was consistent with the results of Quraishi et al^26^ (2020), who found that radiologists around the world were pleased with their teleradiology experience during the COVID-19 crisis.In Africa, a teleradiology system was used to overcome the effects of the COVID-19 pandemic during the lockdowns and to address the lack of adequate staff and expertise.^27^ Similarly, Romanick-Schmiedl and Raghu (2020) indicated that teleradiology is a useful tool during crises and pandemics to maintain the equality of healthcare delivery, allow for remote consultation, and monitor patients with chronic disease.^28^

The shortage of radiologists is a factor with the potential to significantly influence the turnaround time of imaging reports, which appears to be a long-standing problem and is identified several years earlier in the literature.^29^ In this study, the majority of the study participants believed that teleradiology helps radiologists to cope with peak workloads and maximize productivity. This was in line with several studies that found that teleradiology plays a role in balancing workload.^8,19,20^ This was supported by Martin-Noguerol et al^15^ (2021), who referred to the role of teleradiology in increasing productivity in clinical practice and other areas such as administration, education, research, safety, and optimizing policies and protocols. Off-site work can reduce cumulative commuting time, which may be beneficial in terms of minimizing waiting time, reducing workload during on-call hours, and increasing work productivity among radiologists.^25,30^ The results showed a significant association between hospital type and the overall participants’ responses on the role of teleradiology during COVID-19. Those who work in public hospitals showed a greater level of agreement compared to their peers from other hospitals. This may indicate the active role of the MOH in Saudi Arabia in order to integrate teleradiology platforms in their hospitals to improve the quality of service delivery in radiology departments during the COVID-19 pandemic.^6^

Since radiology has played an important role during the COVID-19 pandemic with the use of x-ray and computed tomography to assess patients’ lungs, it was necessary to protect radiologists, radiographers, and nursing staff from being infected.^24^ A large number of the study participants believed in the role of a teleradiology system in reducing the risk of exposure to the COVID-19 virus. A similar result was reported by Shi et al^31^ (2021). The use of a teleradiology system allows for the preservation of a group of radiologists on-site, while the rest safely work off-site from home via a teleradiology system to minimize the potential risk of infections.^16^ Moreover, although half of the study participants believed that the IT has an important role in enabling teleradiology to work successfully during COVID-19, 43% of the participants held the opposite view. With the advent of the COVID-19 pandemic, all governments and healthcare providers shifted some non-essential work to off-site locations. However, in some countries, it has been difficult to fully transition to telework, as it requires highly advanced IT infrastructure. Therefore, it is imperative to notice that optimal IT is required to expedite easy access to patients’ clinical data by radiologists. This is essential for ensuring the quality of the services provided, particularly in terms of accurate and timely diagnosis.^15^

The accuracy of reports issued by radiologists via teleradiology remains a recurrent concern. Compared to in-house reporting, those who use the teleradiology system may not have access to the clinical data or previous examinations that can assist radiologists in image interpretation and impact the quality of radiology reports.^32^ Several studies have discussed the link between a lack of clinical information and the diagnostic accuracy of reports.^33,34^ Nevertheless, Storjohann et al^32^ (2021) found that the absence of clinical information did not negatively impact the accuracy of radiological reports. In this study, 41% of Saudi radiologists seemed to believe that teleradiology has increased the quality of radiology reports during the pandemic, while the rest of the participants expressed uncertain views. This uncertainty raises a critical question concerning the current statutes of Saudi healthcare IT and warrants further investigation regarding IT infrastructure and the integration of patients’ information.

Additionally, the most important benefit of the teleradiology system is the ability to seek a second opinion with subspecialty expertise.^5^ Around 35% of the study participants indicated the possibility of working in a collaborative network via teleradiology during the COVID-19 pandemic as an attractive feature for diagnostic purposes. While that immediate availability of radiological reports regardless of the time is appealing, some radiologists perceive teleradiology as problematic due to the lack of communication with clinicians.^35^ A lack of trust into reports issued by teleradiology was also found due to the lack of interactivity with the clinicians while discussing patients’ images.^32^ However, so-called virtual consultation can be used to improve communication levels and promote interprofessional trust, where distantly located radiologists and clinicians can discuss cases in real time using a computer-screen-share system.^35,36^

### Study limitations

This study was designed to explore radiologists’ perceptions of the role of teleradiology during the COVID-19 pandemic in radiology departments across Saudi Arabia. Therefore, study results were limited to the opinions of Saudi radiologists, and their experiences may not be fully representative of those who work in other countries. Since the panel of respondents was dominated by clinical radiologists (n=87) compared to academicians (n=15), the finding of a correlation between participants’ responses and professional degrees should be interpreted with caution. Although the difference between the pairs of groups in this study could be acceptable, attention needs to be paid to the results due to the small sample size, and future research with a larger sample size is recommended. Additionally, the study collected opinions concerning teleradiology’s suitability for all examinations without specifying the type. Therefore, a good recommendation for future research would be to assess participants’ opinions on which types of imaging examinations that are appropriate to be reported by teleradiology (such as contrast-enhanced or non-contrast-unenhanced). Understanding the current status of teleradiology in Saudi Arabia from radiologists’ perspectives may assist leadership and policymakers in improving the current status of radiology departments and provide guidance to member societies, directors of healthcare facilities, and governments on the requirements of teleradiology services. In addition, further research is required based on an upper-level administrative perspective, which can add huge value by providing a deeper understanding of the use of teleradiology in the future.

In conclusion, teleradiology has played an important role during the COVID-19 pandemic. In order to maintain social distancing and reduce cross-infection, activities at radiology departments have been adapted to remote workstations using teleradiology. The most significant benefit is that teleradiology makes extensive use of technology to raise provider productivity, accelerate the image interpretation process, and improves healthcare services, which reflect a positive impact and suggest that teleradiology could have a future in developing new projects in Saudi hospitals. However, radiologists in the present study were concerned that teleradiology will not be able to perform and interpret intricate cases that require the presence of patients. Concerns were also reported regarding its negative impacts on communication and the discussion of images with other healthcare professionals.
